# Axillary Nodal Involvement in Microinvasive Breast Cancer: A Systematic Review and Meta-Analysis of Sentinel Lymph Node Positivity and Disease Burden

**DOI:** 10.7759/cureus.112510

**Published:** 2026-07-12

**Authors:** Hunter W Brady, Christiana Coonradt, Monica Roberts, Jasneet Gill, Gregory Keagy

**Affiliations:** 1 Medicine, Lincoln Memorial University DeBusk College of Osteopathic Medicine, Knoxville, USA; 2 Internal Medicine, Tennova North Knoxville Medical Center, Knoxville, USA; 3 Surgery, Lincoln Memorial University DeBusk College of Osteopathic Medicine, Knoxville, USA

**Keywords:** axillary de-escalation, axillary staging, meta-analysis, microinvasive breast cancer, nodal me-tastasis, sentinel lymph node biopsy, systematic review, t1mi

## Abstract

Microinvasive breast cancer represents a transitional entity between ductal carcinoma in situ and invasive disease, with generally favorable outcomes but an uncertain risk of lymph node involvement. The role of sentinel lymph node (SLN) biopsy in this population remains controversial. This study aimed to estimate the pooled rate of SLN positivity and characterize the burden of nodal metastasis in patients with microinvasive breast cancer. A systematic review and single-arm meta-analysis were conducted in accordance with PRISMA guidelines. PubMed was searched for studies published between 2015 and 2025 reporting SLN biopsy outcomes in patients with histologically confirmed microinvasive breast cancer (T1mi, ≤1 mm stromal invasion) undergoing sentinel lymph node biopsy. Eligible studies reported the number of patients undergoing biopsy and the number with positive nodes. Random-effects models were used to estimate pooled proportions of SLN positivity and metastatic burden, including isolated tumor cells (ITCs), micrometastases, and macrometastases. Seven studies comprising 11,243 patients were included. Overall, 500 patients (4.4%) were SLN-positive, with a pooled positivity rate of 8.6% (95% CI, 4.3-15.4%) and substantial heterogeneity (I² = 97%). Among SLN-positive cases, pooled proportions were 42.3% (95% CI, 29.2-56.5%) for ITCs, 41.4% (95% CI, 31.4-52.1%) for micrometastases, and 28.9% (95% CI, 15.5-47.5%) for macrometastases. SLN positivity in microinvasive breast cancer is uncommon but not negligible and is most often characterized by low-volume disease. Low-volume disease, including ITCs and micrometastases, comprised the majority of positive nodes, with macrometastases identified in a clinically meaningful subset. These findings support a selective, risk-adapted approach to axillary staging, balancing the potential diagnostic benefit of biopsy against procedural morbidity.

## Introduction and background

Breast cancer remains one of the most diagnosed malignancies worldwide, with approximately 2.3 million new cases reported annually. As detection has improved, surgical management has focused on maintaining oncologic safety while reducing treatment-related morbidity. Advances in breast cancer detection have led to more frequent diagnosis at earlier stages, including lesions that fall along the spectrum between ductal carcinoma in situ (DCIS) and invasive breast cancer [1]. One such entity is microinvasive breast carcinoma, classified as T1mi, which accounts for approximately one percent of all breast cancers. T1mi is defined by invasion measuring ≤1 mm in greatest dimension and typically arises in association with extensive DCIS [2-4].

Microinvasive breast carcinoma is categorized as an invasive disease; however, its biological behavior and clinical outcomes seem to differ from those of larger invasive tumors. Several studies have shown that long-term outcomes for patients with T1mi are excellent and more closely resemble those of DCIS than small invasive cancers, with low rates of recurrence and breast cancer-specific mortality [2,4-7]. Despite these favorable outcomes, the presence of stromal invasion may introduce the potential for lymphatic spread, thus, raising important questions regarding the appropriate approach to axillary staging in this population.

Sentinel lymph node biopsy (SLNB) is widely accepted as the standard of care for invasive breast cancer and provides important prognostic information, but SLNB is not routinely recommended for patients with DCIS due to the low probability of nodal metastasis [7]. Therefore, the role of SLNB in microinvasive breast carcinoma is controversial. Studies have reported sentinel lymph node positivity rates that are generally low, yet highly variable, with reported rates ranging widely across institutional series and population-based analyses [8-10]. When nodal metastases are identified, they are typically of limited metastatic burden, including isolated tumor cells (ITCs) or micrometastases, though macrometastatic involvement has also been documented in a subset of patients [5].

While there is growing interest in axillary management for T1mi, the literature is limited by heterogeneity in the use of immunohistochemistry as well as variability in patient selection and reporting of metastatic burden. These limitations have resulted in a lack of consensus regarding the incidence and clinical significance of sentinel lymph node positivity in microinvasive breast carcinoma, and there is uncertainty surrounding which patients may derive meaningful benefit from SLNB [11]. As a result, clinical practice is inconsistent, and the decision to perform SLNB is individualized without robust pooled evidence to guide recommendations.

Given this, a comprehensive study of available data is needed to quantify the incidence and burden of sentinel lymph node metastasis in microinvasive breast carcinoma. To address this gap, we performed a systematic review and single-arm meta-analysis to evaluate sentinel lymph node positivity rates among patients with T1mi. The objective of this study is to provide a more accurate estimate of nodal involvement in microinvasive breast cancer and to help inform evidence-based, risk-adapted decision-making regarding the use of sentinel lymph node biopsy in this specific patient population.

## Review

Methods

This study was conducted as a systematic review and single-arm meta-analysis to determine the pooled rate of sentinel lymph node positivity in patients with T1mi. The review followed the Preferred Reporting Items for Systematic Reviews and Meta-Analyses (PRISMA) guidelines [12]. This study was not prospectively registered; a protocol was developed a priori and is available upon request.

Literature Search Strategy

A systematic search of the PubMed database was conducted in November 2025 to identify studies that contained information regarding SLNB results in microinvasive breast cancer. The following combinations of keywords using Boolean operators to identify potentially eligible studies were used: (microinvasive breast cancer[Title/Abstract] OR T1mi[Title/Abstract] OR microinvasion[Title/Abstract] OR microinvasive carcinoma[Title/Abstract]) AND (sentinel lymph node[Title/Abstract] OR SLNB[Title/Abstract] OR sentinel node biopsy[Title/Abstract] OR axillary staging[Title/Abstract]).

Population, Intervention, Comparison, and Outcomes (PICO) Framework

Eligibility criteria for this meta-analysis were established based on population, intervention, comparison, and outcome (Table 1) [13].

**Table 1:** Population, Intervention, Comparison, and Outcomes for Study Inclusion in the Meta-Analysis Population, intervention, comparison, and outcomes for systematic review and meta-analysis of sentinel lymph node biopsy outcomes in microinvasive breast cancer.

Component	Definition
Population	Adult patients with histologically confirmed microinvasive breast carcinoma (T1mi; ≤1 mm stromal invasion) who underwent sentinel lymph node biopsy
Intervention	Sentinel lymph node biopsy (SLNB) for axillary staging
Comparator	Not applicable (single-arm meta-analysis; no comparator group)
Outcome	Primary: pooled rate of sentinel lymph node positivity (presence of any nodal metastasis); secondary: distribution of metastatic burden among SLN-positive patients, categorized as isolated tumor cells (ITCs; ≤0.2 mm), micrometastases (0.2-2.0 mm), and macrometastases (>2.0 mm)

Study Selection

All titles, abstracts, and full-text articles were screened by two reviewers independently. Discrepancies were resolved through discussion. Studies were assessed against predefined inclusion and exclusion criteria. Studies were eligible for inclusion if they evaluated patients with histologically confirmed microinvasive breast carcinoma (defined as T1mi on final surgical pathology) and reported SLNB outcomes. Included studies were required to provide the number of T1mi patients who underwent SLNB and the number who were SLN-positive. When available, the burden of nodal metastasis, classified as ITCs, micrometastasis, or macrometastasis, was also extracted. Eligible study designs included retrospective or prospective cohort studies and registry-based analyses published within the past 10 years (2015-2025) to minimize heterogeneity in methodologies and detection techniques.

Studies were excluded if they did not specifically investigate microinvasive breast carcinoma, did not include patients undergoing SLNB, or lacked numerical SLNB results. Review articles, editorials, preclinical research, conference abstracts, and case reports were also excluded. Study selection was performed in multiple rounds to ensure accuracy and consistency with the predefined eligibility criteria.

Data Extraction and Statistical Analysis

Data extraction was performed by two reviewers using a standardized data extraction form. Extracted data were cross-checked for internal consistency prior to analysis. Data collected from eligible studies included the number of microinvasive patients, the number undergoing SLNB, and the number of SLNB-positive cases. When available, data detailing disease burden classified as ITCs (≤0.2 mm), micrometastasis (0.2-2.0 mm), and macrometastasis (>2.0 mm) were obtained.

The primary effect measure was the proportion of patients with T1mi who had a positive SLNB, as defined by the presence of any metastatic disease within the sentinel lymph node, with corresponding 95% confidence intervals. Secondary outcomes included the distribution of metastatic burden among SLN-positive patients, categorized as ITCs, micrometastasis, and macrometastasis, when such stratified data were reported. Additional variables extracted included study year, country of origin, study design, total number of T1mi patients undergoing SLNB, and reported SLNB positivity rates.

Random-effects meta-analyses of proportions were performed using the meta package in R version 4.5.1 (R Foundation for Statistical Computing, Vienna, Austria) [14]. Pooled proportions were estimated with the inverse-variance method using a logit transformation of proportions, and between-study variance was estimated with the DerSimonian-Laird estimator [15]. Clopper-Pearson confidence intervals were used for study-level proportions [16]. Random-effects models were specified for all pooled analyses, and prediction intervals were generated when applicable. Forest plots displayed study-specific proportions, 95% confidence intervals, random-effects weights, and heterogeneity statistics, including I², τ², and the Cochran Q test p-value. A leave-one-out sensitivity analysis was performed to evaluate the influence of individual studies on the overall pooled estimate and heterogeneity. Extracted data and analysis code are available from the corresponding author upon reasonable request.

Risk of Bias Assessment

Risk of bias was assessed independently by two reviewers using the Risk of Bias in Non-randomized Studies of Interventions (ROBINS-I) tool [17]. Discrepancies between reviewers were resolved through discussion until consensus was reached. Studies were categorized as low, moderate, serious, or critical risk of bias based on the overall domain rating. A traffic-light visualization plot was generated using the Robvis tool for risk-of-bias visualization [18]. Assessment of publication bias using funnel plots or statistical tests was not performed due to fewer than 10 included studies. No formal assessment of the certainty of evidence was performed.

Results

Study Selection and Study Characteristics

**Figure 1:**
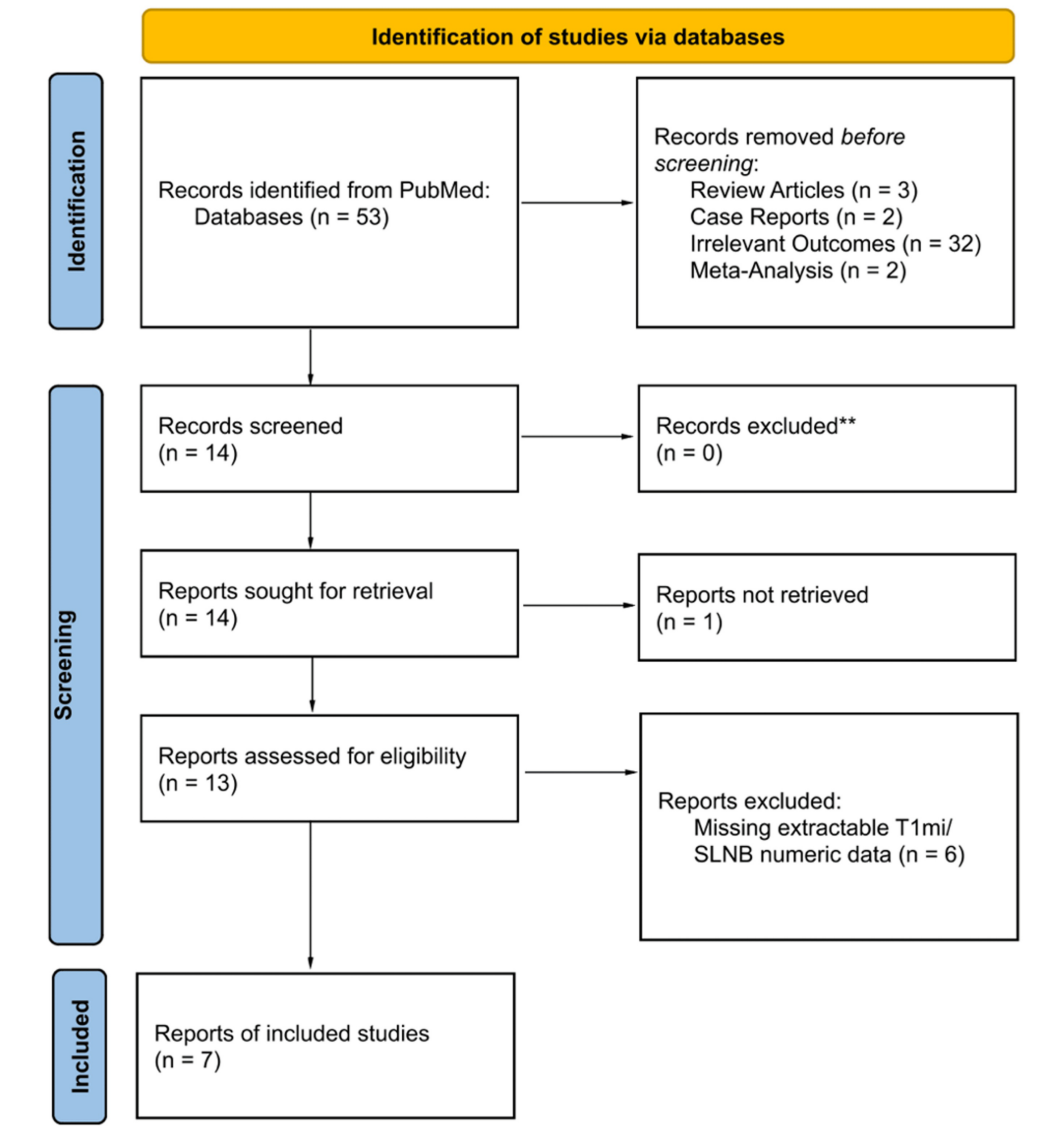
Preferred Reporting Items for Systematic Reviews and Meta-Analyses 2020 flow diagram illustrating the identification, screening, eligibility assessment, and inclusion of studies in the meta-analysis

Initial PubMed search revealed 53 studies for screening. Seven studies met the inclusion criteria and were included for analysis. Figure 1 demonstrates the PRISMA flowchart for study selection. Studies represented geographically diverse cohorts from the United States, Italy, Denmark, and China. Overall, 11,243 patients with T1mi underwent SLNB. Five studies provided additional metastatic burden stratification by ITCs, micrometastasis, and macrometastasis. Full study characteristics are available in Table 2.

**Table 2:** Characteristics of Included Studies Summary of included study characteristics, design, and sentinel lymph node biopsy outcomes in patients with microinvasive breast carcinoma (T1mi). SLN+ rate reflects the proportion of patients with any nodal metastasis identified on sentinel lymph node biopsy. NCDB, National Cancer Database; CBCSG001, Chinese Breast Cancer Sentinel Node Study; DCIS, ductal carcinoma in situ; DCISM, ductal carcinoma in situ with microinvasion; SLN, sentinel lymph node; SLNB, sentinel lymph node biopsy; T1mi, microinvasive breast carcinoma.

Study	Country	Design	Total T1mi Patients with SLNB	SLN+ Rate (%)	Notes
Magnoni et al., 2019 [3]	Italy	Retrospective single-institution	251	12.1	Long-term follow-up (median 11 years)
Fan et al., 2020 [5]	USA	Retrospective (NCDB)	2,609	2.9	Large national cohort; ITC classified as negative SLN
Sun et al., 2015 [11]	China	Retrospective multi-center (CBCSG001)	100	11	Part of CBCSG001 trial; included both DCIS and DCISM patients
Holm-Rasmussen et al., 2019 [8]	Denmark	Nationwide registry	233	21.5	Danish Breast Cancer Group database; detailed non-SLN data
Strang et al., 2020 [1]	USA	Retrospective single-institution (matched)	125	7	Matched 1:1:1 with pure DCIS and small invasive cancers
Orzalesi et al., 2016 [4]	Italy	Retrospective single-institution	126	14.3	Florence cohort; focused on axillary staging subset (2000-2014)
Gooch et al., 2019 [9]	USA	Retrospective (NCDB)	7,803	3.9	Nomogram development study

Overall SLNB Positivity in T1mi

**Figure 2:**
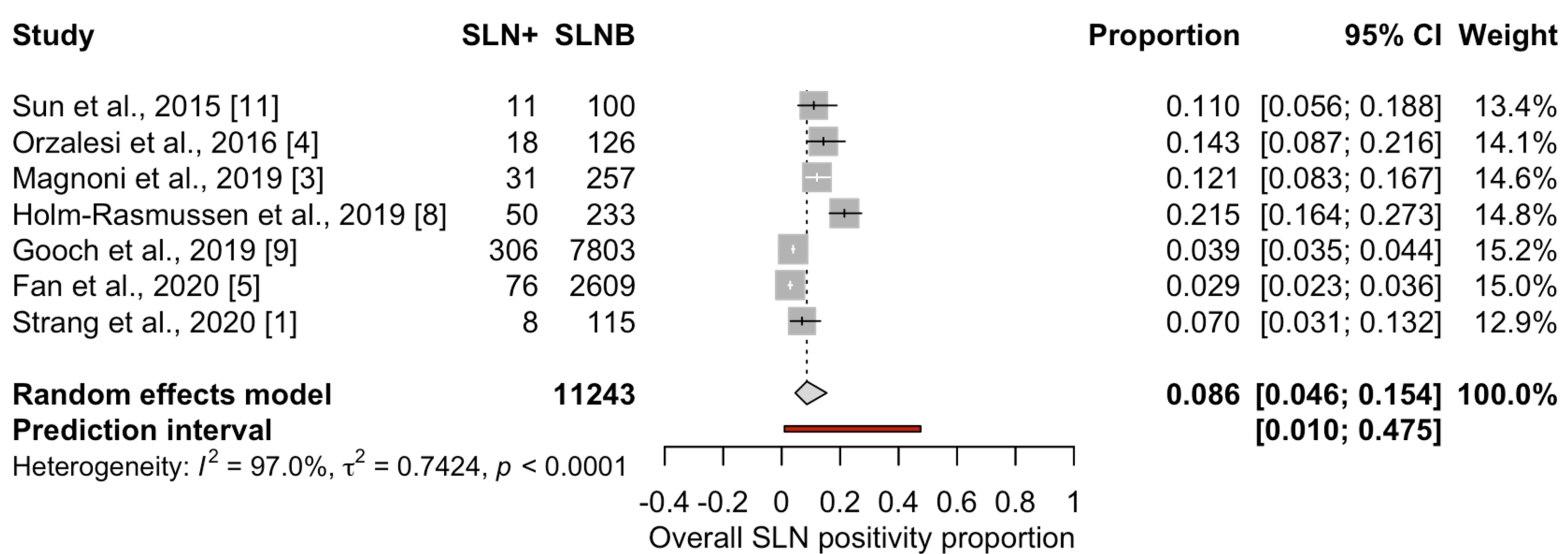
Forest plot showing the proportion of sentinel lymph node positivity across included studies in T1mi breast cancer The diamond represents the overall pooled estimate using a random-effects model (8.6%, 95% CI: 4.6-15.4%). Heterogeneity statistics: I2 = 97%, τ2 = 0.7424, p < 0.0001

Across all included studies, 500 of 11,243 patients (4.4%) were SLN-positive, with reported rates ranging from 2.9% to 21.5%. Random-effects meta-analysis demonstrated a pooled SLN positivity rate of 8.6% (95% CI, 4.6-15.4%) (Figure 2). Heterogeneity was substantial (I² = 97%), likely reflecting wide differences in sample size, pathological protocols, and detection methods across studies.

A leave-one-out meta-analysis demonstrated that the pooled SLN positivity estimate was stable (range 7-11%) (Table 3). Excluding large U.S. based studies, Fan et al. and Gooch et al., a modest increase in the pooled estimate (10-11%) was observed [5,9]. Conversely, removing smaller studies slightly decreased the overall SLN positivity.

**Table 3:** Leave-one-out sensitivity analysis of the pooled sentinel lymph node positivity rate in patients with T1mi breast cancer Each row shows the pooled SLN positivity proportion and heterogeneity statistic (I²) obtained when the indicated study is excluded from the meta-analysis.

Study	Pooled Proportion % (95% CI)	Heterogeneity (I²)
Magnoni et al., 2019 [3]	0.08 (0.04-0.15)	97.20%
Fan et al., 2020 [5]	0.10 (0.05-0.21)	97.10%
Sun et al., 2015 [11]	0.08 (0.04-0.16)	97.40%
Holm-Rasmussen et al., 2019 [8]	0.07 (0.04-0.12)	94.30%
Strang et al., 2020 [1]	0.09 (0.05-0.17)	97.50%
Orzalesi et al., 2016 [4]	0.08 (0.04-0.15)	97.20%
Gooch et al., 2019 [9]	0.10 (0.04-0.210)	96.60%

Metastatic Burden Among SLN-Positive Patients

**Figure 3:**
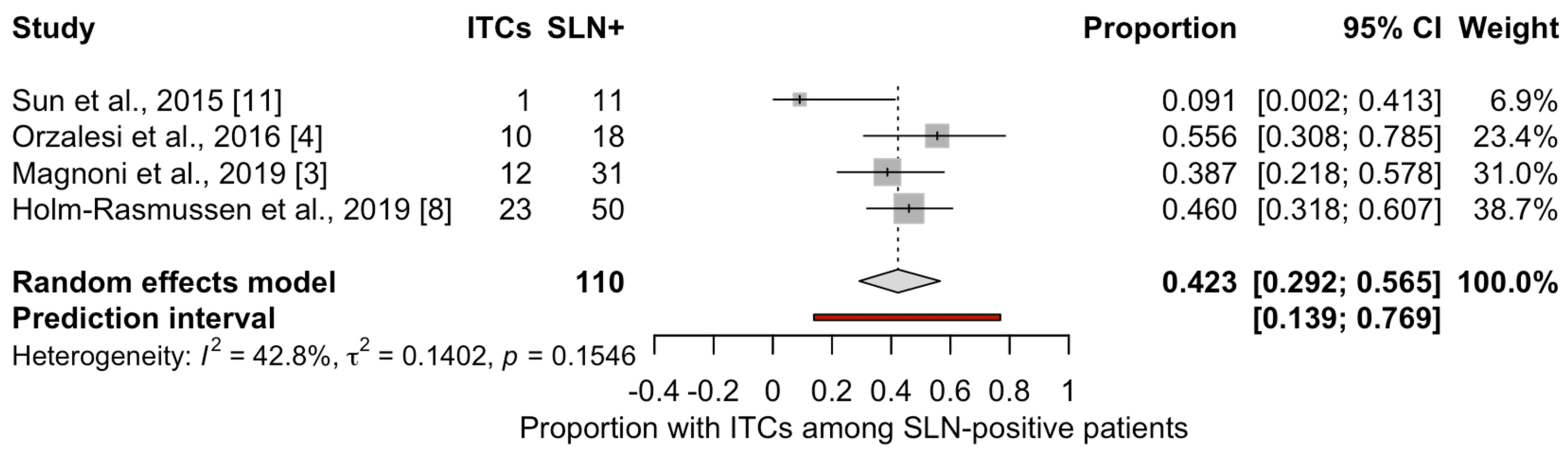
Forest plots showing the pooled proportions of isolated tumor burden among sentinel lymph node-positive patients with microinvasive (T1mi) breast cancer using random-effects models Isolated tumor cells (ITCs). Pooled proportion 42.3% (95% CI: 29.2-56.5%). Heterogeneity: I2 = 42.8%, τ2 = 0.1402, p = 0.1546

Four studies (n = 110 SLN-positive cases) reported stratification of ITCs. ITC rates ranged from 9% to 56%, and meta-analysis yielded a pooled ITC proportion of 42.3% (95% CI, 29.2%-56.5%) (Figure 3). Moderate heterogeneity was observed (I² = 42.8%).

**Figure 4:**
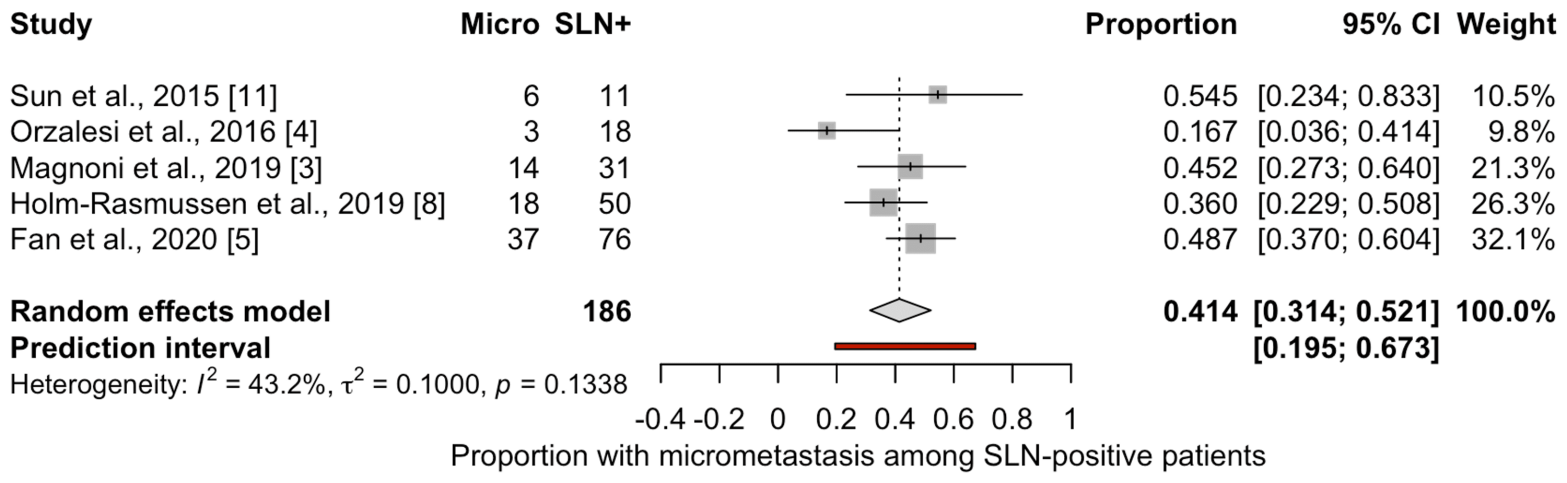
Forest plots showing the pooled proportions of micro-metastatic burden among sentinel lymph node-positive patients with microinvasive (T1mi) breast cancer using random-effects models Micrometastases (0.2-2 mm). Pooled proportion 41.4% (95% CI: 31.4-52.1%). Heterogeneity: I2 = 43.2%, τ2 = 0.1000, p = 0.1338

Five studies (n = 186 SLN-positive cases) reported micrometastasis. Reported micrometastasis rates ranged from 17% to 55%, and the pooled micrometastasis proportion was 41.4% (95% CI, 31.4-52.1%) (Figure 4), with moderate heterogeneity (I² = 43.2%).

**Figure 5:**
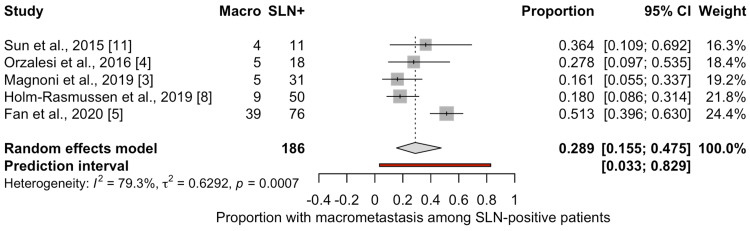
Forest plots showing the pooled proportions of macro-metastatic burden among sentinel lymph node-positive patients with microinvasive (T1mi) breast cancer using random-effects models Macrometastases (>2 mm). Pooled proportion 28.9% (95% CI: 15.5-47.5%). Heterogeneity: I2 = 79.3%, τ2 = 0.6292, p = 0.0007

Across the same five studies, macrometastasis rates ranged from 16% to 51%. Random-effects meta-analysis yielded a pooled macrometastasis proportion of 28.9% (95% CI, 15.5-47.5%) (Figure 5), with high heterogeneity (I² = 79.3%).

Risk of Bias Assessment

**Figure 6:**
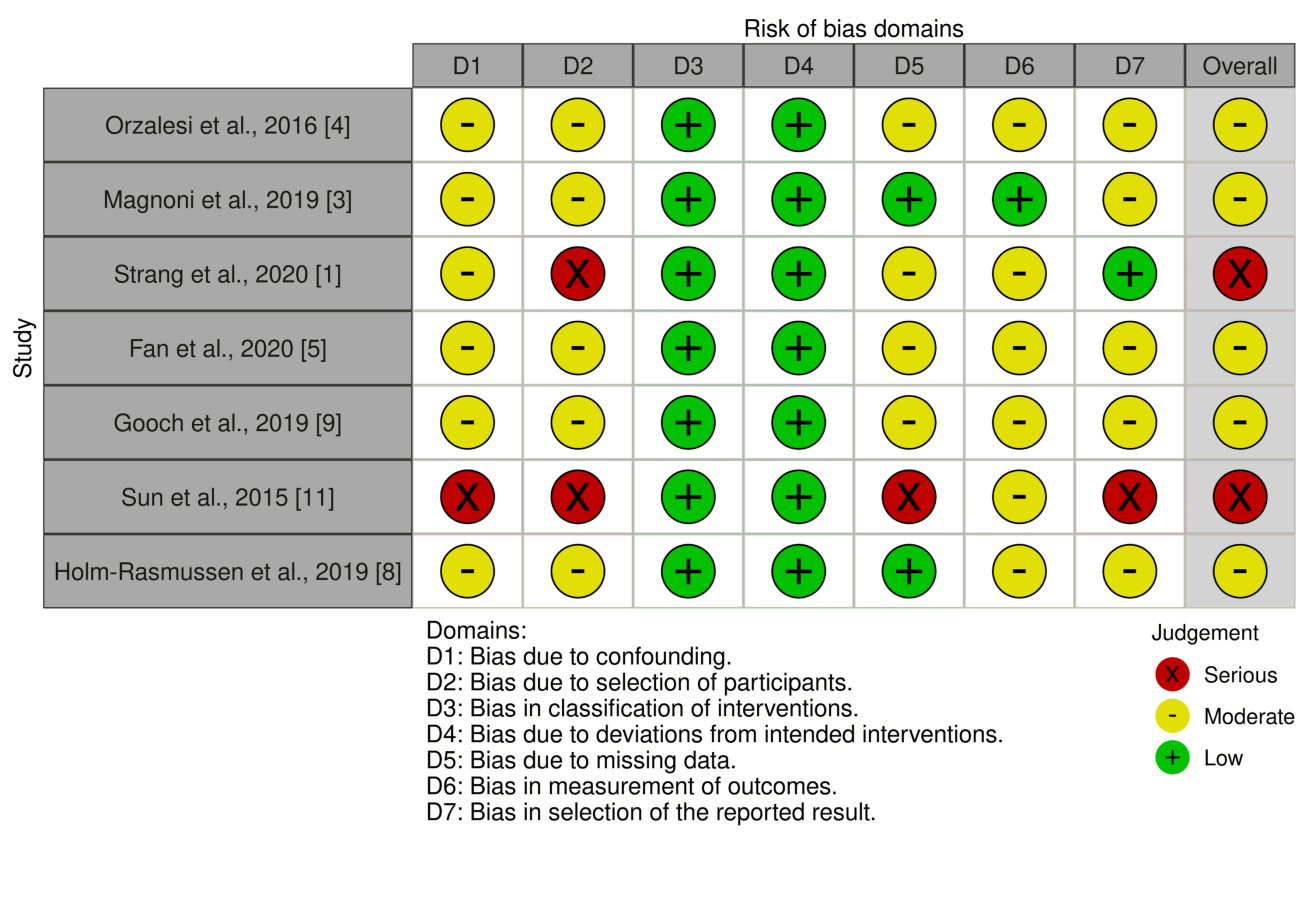
Traffic light plot showing the risk of bias assessment of the seven included studies using the ROBINS-I (Risk of Bias in Non-randomized Studies of Interventions) tool

**Figure 7:**
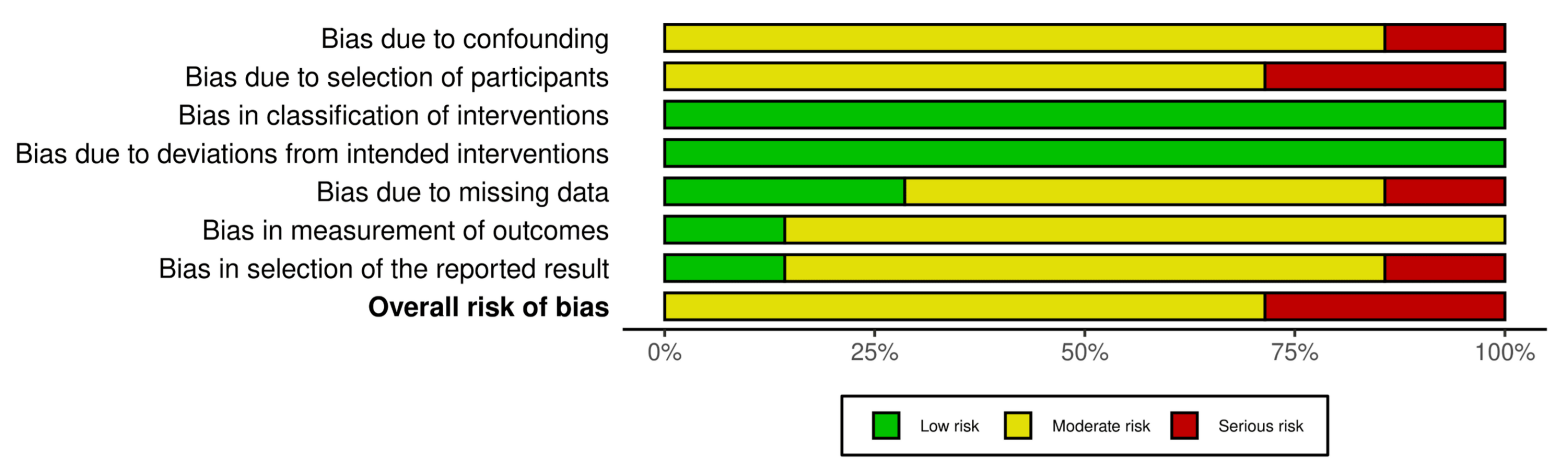
Summary plot showing the risk of bias assessment of the seven included studies using the ROBINS-I (Risk of Bias in Non-randomized Studies of Interventions) tool

Seven domains were evaluated: bias due to confounding (D1), bias in selection of participants into the study (D2), bias in classification of interventions (D3), bias due to deviations from intended interventions (D4), bias due to missing data (D5), bias in measurement of outcomes (D6), and bias in selection of the reported result (D7). The results of this assessment are summarized in Figures 6-7.

The overall risk of bias was rated as moderate in five studies and serious in two studies. The moderate overall rating in the majority of studies was primarily driven by a moderate risk of bias due to confounding (Domain 1), which was present in all studies. As per ROBINS-I guidance, moderate risk in Domain 1 typically resulted in an overall moderate rating. Two studies (Strang et al. and Sun et al.) were judged to have an overall serious risk of bias, mainly due to serious risk in participant selection (D2) and, in one case, additional concerns regarding missing data and selective reporting.

The moderate risk of bias due to confounding reflects the observational, non-randomized nature of all included studies. Residual confounding and confounding by indication (particularly patient selection for sentinel lymph node biopsy) could not be fully excluded, even in analyses that performed multivariable adjustment.

No study demonstrated a critical risk of bias in any domain. Outcome ascertainment was objective (histopathological nodal status), definitions of microinvasive breast cancer were consistent, and there was no strong evidence of selective outcome reporting. Missing data were generally low to moderate, although more pronounced in one smaller database-derived study.

Discussion

In this meta-analysis of more than 11,000 patients with T1mi, the pooled SLN positivity rate was 8.6% (95% CI, 4.6-15.4%), with macrometastases observed in 28.9% of positive nodes (pooled proportion 28.9%; 95% CI, 15.5-47.5%). These rates indicate a measurable but relatively low frequency of nodal involvement in T1mi. Although microinvasive carcinoma is defined by ≤1 mm of stromal invasion, the included studies and prior reports show that nodal metastases can occur in a subset of patients, even though the overall rate remains relatively low [3,5].

Across the included studies within this meta-analysis, low-volume metastases such as ITCs and micrometastases were the most frequently detected SLN abnormalities [2,3,5]. However, the identification of macrometastatic disease in both our pooled dataset and prior large series studies suggests that nodal positivity in T1mi cannot be dismissed simply as an artifact or displaced epithelium [9-10]. Several nationwide cohort studies and prior meta-analyses have reported macrometastases in a small but measurable proportion of patients with T1mi, consistent with the findings of this analysis [3].

The substantial statistical heterogeneity observed for the primary endpoint (I² = 97%) likely reflects several identifiable differences among the included studies rather than random variation alone. Study size spanned roughly 78-fold, from 100 to 7,803 patients, meaning the large national cohorts (Fan et al. and Gooch et al.) and the smaller single-institution series contributed very different information to the pooled estimate [5,9]. Classification of low-volume disease also differed: ITCs were counted as node-negative in one national database cohort (Fan et al.) but as positive nodal disease in other series, which directly shifts the reported positivity rate [5,9]. Additionally, other differences between nationwide registry-based analyses versus single-institution cohorts and variation in immunohistochemistry use and pathologic protocols for detecting and measuring nodal metastases may contribute to the substantial heterogeneity observed. Given the small number of included studies (n = 7), formal meta-regression to quantify these effects is statistically underpowered and was not performed; instead, the leave-one-out sensitivity analysis (Table 3) demonstrated that the pooled estimate remained stable (range 7-11%), indicating that no single study drove the overall result despite the observed heterogeneity.

Despite the measurable rate of SLN involvement, long-term outcomes for patients with microinvasive breast cancer remain excellent [19]. This mirrors the survival observed in pure DCIS and is substantially better than the outcomes seen in small invasive cancers [1,3,4]. Several studies have shown no significant difference in recurrence or survival between SLN-positive patients who did or did not undergo full axillary node dissection, reinforcing that SLNB can provide staging information but is not necessary for clinical treatment decisions [5]. Even when there is SLN positivity present, low metastatic burden and favorable disease biology appear to mitigate the prognostic impact of nodal involvement [4,11].

In the context of the broader movement toward axillary de-escalation, these observations are further informed by the SOUND (Sentinel Node vs Observation After Axillary Ultrasound) trial. This randomized phase three noninferiority study demonstrated that omission of axillary surgery was noninferior to sentinel lymph node biopsy (SLNB) for five-year distant disease-free survival (98.0% vs 97.7%) in patients with small (≤2 cm), clinically node-negative breast cancers and negative preoperative axillary ultrasound - despite a 13.7% rate of nodal positivity in the SLNB arm [20]. Our findings in T1mi patients reinforce this trend: SLNB appears to be low-yield overall, yet a small subset with macrometastatic disease exists that would be missed with routine omission. At present, we lack a fully validated, comprehensive model to reliably identify these higher-risk individuals preoperatively.

As we move forward, it is increasingly evident that tumor biology, rather than purely anatomic nodal staging, drives adjuvant treatment decisions and long-term outcomes in early-stage disease. Factors such as hormone receptor status, HER2 expression, grade, and genomic assays are assuming greater importance, potentially rendering the incremental information from SLNB even less clinically consequential in many T1mi cases. Our analysis contributes to the growing body of literature attempting to define optimal candidates for de-escalation of axillary surgery, a challenge faced across breast cancer subtypes today. Potential risk features (e.g., younger age, HER2 positivity, high-grade or extensive DCIS components, and larger associated ductal elements) warrant further prospective validation, ideally through nomogram-based tools or integration with preoperative imaging and multidisciplinary assessment [2-3,6,9-10].

Optimal management of the axilla in T1mi therefore favors a selective, individualized, and risk-adapted approach. SLNB may be most appropriate for patients with high-risk pathological features or those needing mastectomy where delayed staging isn’t feasible [3,11]. In contrast, it appears to be reasonable to forgo SLNB in patients with low-risk disease undergoing breast-conserving surgery [3,4,10], particularly when aligned with SOUND trial eligibility criteria (e.g., negative axillary ultrasound and hormone receptor-positive, HER2-negative biology) and when nodal status is unlikely to influence adjuvant therapy recommendations [2-3,8]. Multiple studies emphasize that a multidisciplinary evaluation is essential for aligning treatment decisions with individual risk and values [2-3,10]. This should include integrating preoperative imaging, histopathology, and keeping patient preference as a top priority [2-3,10].

This review has several limitations. First, the literature search was restricted to a single database (PubMed); although the full Boolean strategy is disclosed to support replication, reliance on one database may have missed studies indexed elsewhere. Second, all included studies were retrospective and observational, introducing the potential for residual confounding and confounding by indication, particularly in the selection of patients for sentinel lymph node biopsy. Third, the included studies varied in design, pathology and immunohistochemistry protocols, classification of low-volume nodal disease, and reporting of metastatic burden, contributing to the substantial heterogeneity discussed above and limiting the ability to draw definitive conclusions. Fourth, diversity in SLNB indications and institutional practice patterns may limit generalizability. Fifth, limited patient-level detail precluded analysis of whether biologic subtype or the extent of the associated DCIS component influenced SLN positivity or outcomes. Finally, the review was not prospectively registered.

Future work should prioritize prospective evaluation of microinvasive disease, standardizing definitions and pathology for microinvasion, and the development of validated prediction models capable of identifying high-risk individuals. Additional research on molecular differentiation between DCIS, T1mi, and small invasive cancers may also help clarify the biological drivers of metastasis in this unique and underrepresented population.

## Conclusions

This meta-analysis demonstrates that nodal involvement in T1mi occurs at a measurable but relatively low overall rate, with low-volume disease comprising the majority of positive cases. Given the generally favorable prognosis of T1mi and the heterogeneous distribution of metastatic burden, these results support a selective, individualized, risk-adapted approach to sentinel lymph node biopsy in this population. Future research should prioritize prospective evaluation, standardization of microinvasion definitions and pathology protocols, and development of validated risk-stratification tools to further refine axillary management while minimizing procedural morbidity.
